# Keeping Allergen Names Clear and Defined

**DOI:** 10.3389/fimmu.2019.02600

**Published:** 2019-11-19

**Authors:** Sanny K. Chan, Anna Pomés, Christiane Hilger, Janet M. Davies, Geoffrey Mueller, Annette Kuehn, Andreas L. Lopata, Gabriele Gadermaier, Marianne van Hage, Monika Raulf, Richard E. Goodman

**Affiliations:** ^1^Division of Allergy and Immunology, Department of Pediatrics, National Jewish Health, Denver, CO, United States; ^2^INDOOR Biotechnologies, Inc. Charlottesville, VA, United States; ^3^Department of Infection and Immunity, Luxembourg Institute of Health, Esch-sur-Alzette, Luxembourg; ^4^Centre for Children's Health Research, Institute of Health and Biomedical Innovation, Queensland University of Technology, Brisbane, QLD, Australia; ^5^Metro North Hospital and Health Service, Brisbane, QLD, Australia; ^6^National Institute of Environmental Health Sciences, Durham, NC, United States; ^7^Australian Institute of Tropical Health and Medicine, James Cook University, Townsville, QLD, Australia; ^8^Department of Biosciences, University of Salzburg, Salzburg, Austria; ^9^Division of Immunology and Allergy, Department of Medicine Solna, Karolinska Institutet and University Hospital, Solna, Sweden; ^10^Institute of Prevention and Occupational Medicine of the German Social Accident Insurance, Institute of the Ruhr-Universitat Bochum, Bochum, Germany; ^11^Food Allergy Research and Resource Program, Deptartment of Food Science and Technology, University of Nebraska-Lincoln, Lincoln, OR, United States

**Keywords:** allergen nomenclature, WHO/IUIS, taxonomy, diagnostic, airway, food, dermal

## Abstract

The World Health Organization/International Union of Immunological Societies (WHO/IUIS) Allergen Nomenclature Sub-Committee was established in 1986 by leading allergists to standardize names given to proteins that cause IgE-mediated reactions in humans. The Sub-Committee's objective is to assign unique names to allergens based on a critical analysis of confidentially submitted biochemical and clinical data from researchers, often prior to publication to preserve consistency. The Sub-Committee maintains and revises the database as the understanding of allergens evolves. This report summarizes recent developments that led to updates in classification of cockroach group 1 and 5 allergens to animal as well as environmental and occupational allergens. Interestingly, routes, doses, and frequency of exposure often affects allergenicity as does the biochemical properties of the proteins and similarity to self and other proteins. Information required by the Sub-Committee now is more extensive than previously as technology has improved. Identification of new allergens requires identification of the amino acid sequence and physical characteristics of the protein as well as demonstration of IgE binding from subjects verified by described clinical histories, proof of the presence of the protein in relevant exposure substances, and demonstration of biological activity (skin prick tests, activation of basophils, or mast cells). Names are assigned based on taxonomy with the abbreviation of genus and species and assignment of a number, which reflects the priority of discovery, but more often now, the relationships with homologous proteins in related species.

## Introduction

Advances in molecular biology, recombinant protein technology, methods of genomic, and proteomic analysis, structural biology, and high throughput screening have led to a greater understanding of allergenic proteins over the past 30 years. Identification of new allergens and improved characterization dictate the need for revision of some allergens and updated requirements for allergen nomenclature.

In the 1980's key clinical and experimental allergists led by D Marsh at Johns Hopkins University, USA, L Goodfriend (McGill University, Canada), TP King (Rockefeller University, USA), H Lowenstein (University of Copenhagen (Denmark) and TAE Platts-Mills (University of Virginia, USA) framed basic rules for naming allergenic proteins in common allergenic sources (1). Over 2 years they agreed to a strategy of naming allergens based on taxonomy with identification using the first three letters of the genus, the first letter of the species and a number for order of identification. The first allergens were mostly inhalant proteins from pollens of weeds, trees and grasses and a few from animal dander, fungi, and venom proteins from stinging insects. The committee recognized that a systemic standardized nomenclature was needed for consistent identification in scientific publications. The standardized naming of allergens has become a formalized process where allergen names are assigned by the WHO/IUIS Allergen Nomenclature Sub-Committee after a defined submission process that includes data on IgE binding to identified novel target proteins or glycoproteins with the most recent major revision occurring in 2018 (2).

Allergen names are assigned with the first 3 or sometimes 4 letters of the genus, one or sometimes two letters for the species followed by an Arabic numeral, based on order of discovery (2). However, the same Arabic number is often used for conserved protein families in related taxa. Often species have evolved by duplication of genes with mutations including additions or deletion of nucleotides to produce alternative proteins of similar function, resulting in the occurrence of isoallergens as well as isoforms or variants within the individual organism or in related organisms of the same species. The isoallergens are designated by the addition of two digits after the decimal in the number and isoforms or variants by addition of two more digits (e.g., Amb a 1.0101). Since the evolutionary steps involved in generating each new isoform or variant is usually not known, the committee bases designations on the percent identity of amino acids compared to the first identified sequence. Sequences within ~67% identity to the original allergen are designated as isoallergens and sequences differing by <90% identity are isoform or variants.

**Figure 1:**
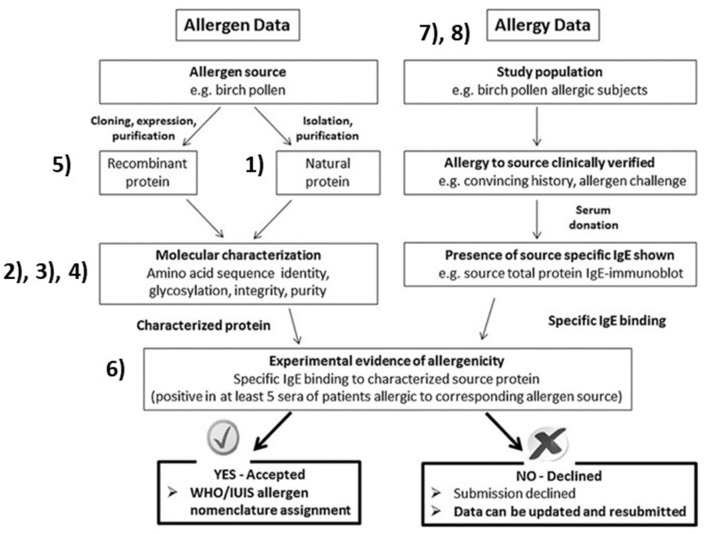
Overall allergen characterization for submission to the WHO/IUIS Allergen Nomenclature Sub-Committee for possible name recognition. Figure with copyright allowance from Pomés et al. (2). Numbers are inserted beside key blocks, identifying topical areas and challenges related to our evaluations. (1) Nearly identical repeated sequences confuse characterization of the natural protein. (2) Amino acid identity matches across distant taxonomic relations are unlikely to represent cross-reactive proteins. (3) Native allergens of co-valently linked independent proteins such as Fel d 1 and Ory 3. (4) Carbohydrate IgE binding epitopes (CCD, alpha-gal) are not (yet) named in this system. (5) Studies testing cloned, recombinant proteins without native protein bridging can leave doubts. (6) Biological evidence, either basophil histamine release or activation, or skin prick test with pure protein. (7) Aeroallergen identities in clinical tests are typically not verified in allergen diagnostic testing due to lack of available components. (8) Occupational and food allergen testing typically involve few study subjects.

Expression of recombinant proteins for IgE tests and characterization has led to more accurate identification. Changes to the nomenclature have attempted to incorporate an increased understanding of allergens. Higher resolution studies and isolation techniques of previously defined allergens now shows some to be composed of multiple subunits from different genes; Fel d 1, or multimers; collagen, vicilins, and glycinins. This manuscript focuses on recent challenges and underlying reasons for name assignment by the WHO/IUIS Nomenclature Sub-Committee. We refer those working on allergen identification to Figure 1, from our 2018 publication with references to specific sections in this paper to explain the ideal process (2). The science of allergen determination has improved since 1990, for characterization of allergenic sources, protein sequences, post-translational modification determination, improved methods for measuring IgE binding, mediator release and measurements of specific bioactivity (skin tests and basophil assays). Scientists striving to characterize allergens should be aware of and using techniques appropriate to the types of allergens including those of foods, contact allergens, aeroallergens and venoms or salivary sources of allergy.

## Challenges for Future Allergen Names

### Repeated Structure of Group 1 Cockroach Allergens Is Not Reflected in Current Allergen Nomenclature

Cockroach allergens from group 1 were difficult to name using the current WHO/IUIS paradigm. Cloning of the DNA encoding for Bla g 1 allergen revealed the existence of multiple DNA repeats encoding approximately 100 amino acids each (3). The molecular structure of Bla g 1 showed that two tandem amino acid repeats form a spherical fold (4). A recent analysis of the *Blatella germanica* genome (5) suggests there are 5 separate gene products, and each produce 1 to 5, partial or full repeats of the Bla g 1 structural motif. In total, there may be 9 other Bla g 1-like allergens, with 60–90% identity to the Bla g 1 structure. Prior to the genomic data, it was unclear whether the different Bla g 1 polymorphisms derived from a different gene. In addition, there was not a precedent for an allergen with this kind of structure and genomic origin.

Besides cockroaches, it is worthwhile to define new rules for assigning a nomenclature to this type of allergen, given the possibility that Bla g 1-like homologs exist in other sources, such as locusts and mealworms, recently included as food sources. Allergens belong to a selected group of protein families (6, 7), and it is likely that any Bla g 1-like homologs in these sources could also be an allergen. Indeed, a homolog of Bla g 1 was noted in the genome of *Locusta migratoria* (8). Further examination reveals that there are two genes with 2 and 3 copies of the Bla g 1-like structure, respectively. Other coleopteran species have been proposed as a substitute protein source in place of mammalian meat. A search of genomes of related species revealed that *Tribolium castaneum*, has one gene with 9 repeats similar to the Bla g 1- structure. This repeated sequence structure is likely to occur in other beetles and there are reported occupational and food allergies reported from a larval beetle (mealworm) of *Tenebrio molitor* (9). Homologs of Bla g 1 can be found in many other insect species as well (10). So far, the protein(s) responsible for reactions to mealworm are not yet identified except for tropomyosin with homology to shrimp proteins. The structural complexity of allergens like Bla g 1 are not reflected in the current allergen nomenclature. For similar cases, the Allergen Nomenclature Sub-Committee discussed the possibility (not yet approved) of using capital letters behind the allergen name to signify either the different genes from which the allergen originated, or different duplexes derived from a gene (i.e., Bla g 1.0101A). Knowledge of the origin of unique allergen structures may help to define a more informative allergen nomenclature for these unusual allergens.

### Group 5 Cockroach Allergens Are Glutathione S-Transferases (GSTs), Consideration of the Degree of Amino Acid Identity

**Figure 2:**
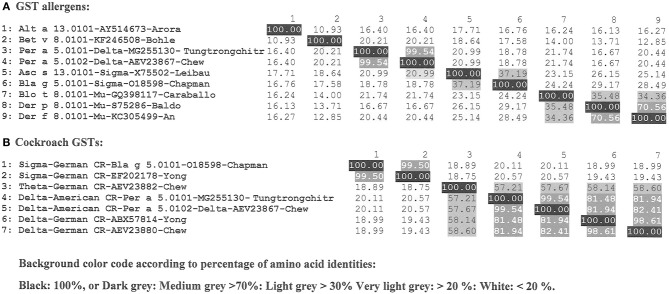
Homologous protein GST allergens are currently categorized as isoallergens with a threshold of 67% amino acid identity although they may belong to different protein classes (sigma, delta, theta, mu). Percent identities are shown. **(A)** Nine groups of GST allergens from different taxa to understand sequence identities across sources. **(B)** Cockroach GSTs within German and American cockroaches with known allergens and genomic sequences with comparison of amino acid sequence identities.

In recent years the WHO/IUIS Sub-Committee has used similar allergen numbers for homologous proteins across related taxonomic groups when possible. Glutathione *S*-transferases (GST) are common enzymes present in multiple organisms from different taxonomic groups. One of the main functions of GSTs is detoxification by catalyzing the addition of a glutathione (GSH) molecules to other commonly toxic compounds that are ultimately removed from the cell (11, 12). Nine groups of allergens are recognized as GSTs and are listed in the WHO/IUIS Allergen Nomenclature database (www.allergen.org), including those from cockroaches (Bla g 5 and Per a 5), mites (Der p 8, Der f 8 and Blo t 8), helminths (Asc s 13, Asc l 13), fungi (Alt a 13), and plants (Bet v 8) (Figure 2A).

In general, homologous allergens from the same species are included in the same allergen group as isoallergens (e.g., Cyn d 1.0101 and Cyn d 1.0201 in Table S1) if they share a suggested threshold of 67% amino acid identity (2). At 67% identity, IgE cross-reactivity is possible. However, naming additional GSTs from cockroach in group 5, has been challenging due to their low sequence identities (down to 18% identity). For those identified as binding IgE, they could be given a different name and number (Figure 2B). We considered these enzymes belong to different protein classes (sigma, delta, theta, mu) as functionally related. The Bla g 5 from German cockroach (*Blattella germanica*) was the first cockroach GST to be identified (O18598), and it is a sigma class GSTs (11). Additional GSTs from German cockroach have been identified, though these have not been submitted to the WHO/IUIS committee with proof of IgE binding: another sigma GST (EF202178), a theta GST (AEV23882) and two delta GSTs (ABX57814 and AEV23880). Two GSTs from the American cockroach (*Periplaneta americana*) were identified as binding IgE, and they belong to the delta class of GSTs [MG255130 (13) and AEV23867] and share 99.54% amino acid identity. When these GSTs were submitted, we decided to include them as Per a group 5 proteins even though their identity to Bla g 5 was only 19% identity with Bla g 5. They were named Per a 5.0101 and Per a 5.0102. If other cockroach GSTs are submitted to the database in the future, they will be assigned as isoallergens if fitting within the 67% identity range to the respective group 5 allergens.

GSTs from other species are and have been assigned to other numbers as seen in Figure 2A (Alt a 13, Bet v 8, Asc a 13, Blo t 8, Der p 8 amd Der f 8. Isoallergens of each of these will be assigned, based on sequence comparison, to currently named members of the respective source species. The committee now prefers to retain primary allergen numbers the same across related taxonomic groups, but that is not always possible. It is important to note that while enzyme functions may be retained with great diversity in amino acid sequences, the ability for IgE to bind across those species and meet the criteria of allergenicity is not retained.

### Covalently Linked Dimeric Allergens and Allergen Nomenclature

Mammalian allergens of the secretoglobin family are heterodimers, encoded by separate genes. Secretoglobins (SCGB) represent a large protein family found in mammals and marsupials. Common characteristics are secretion in many body fluids, small size, alpha-helical, and dimeric structure creating a hydrophobic binding pocket (14, 15). Major allergens from cat and rabbit are Fel d 1 and Ory c 3. Both are glycosylated heterodimers linked by three disulfid bridges (16, 17). Fel d 1 also forms tetramers composed of two heterodimers. Each protein chain of the heterodimer is encoded by a single gene and both are needed to compose the natural molecule and may be needed for achieving full allergenic activity. The current allergen nomenclature system did not foresee linked heteromeric allergens and the names allocation for Fel d 1 and Ory c 3 were not consistent. Whereas, Fel d 1 is listed as Fel d 1.0101 for the dimer, composed of a chain 1 and a chain 2, products of two separate genes (17), Ory c 3 is listed as 2 isoallergens with Ory c 3.A.0101, chain A (lypophillin CL2) and Ory c 3.B.0101, chain B (lypophillin AL). The nomenclature for Ory c 3 suggests that both isoallergens have to be considered together as a full allergen (17). The two proteins do not exist naturally as monomers and allergenicity can not be assessed individually. The Sub-Committee recently renamed the Fel d 1 proteins as Fel d 1.A.0101 for chain 1 and Fel d 1.B.0101 for chain 2, consistent with the rabbit allergen.

Other examples are recently added allergens, barramundi collagen Lat c 6 and salmon allergen Sal s 6 proteins. Collagen is a triple-stranded rope-like coiled structure, composed of 3 protein chains. Each chain has been given an isoallergen name, despite the fact that they do not exist as monomers. Currently it is only possible to evaluate separate IgE binding if using recombinant monomeric proteins and thus it is not possible to assess the allergenicity of each of the entire native proteins. The committee may reevaluate this policy in the future.

### Carbohydrates, New Allergen Epitopes Not Represented in the Allergen Nomenclature Database

Cross-reactive carbohydrate determinants (CCDs) are carbohydrate epitopes carrying an α-1,3-fucose and, or β 1,2 xylose on complex carbohydrates of some glycoproteins are the main targets of IgE recognition. They are present on many plant and insect proteins and responsible for extensive IgE cross-reactivity between different allergen sources (18). However, there is a general consensus that those glycans do not trigger noticeable clinical symptoms. Two other carbohydrate groups have been associated with food allergy and anaphylaxis (19). Galactose-α-1,3-galactose (α-gal), a carbohydrate present on mammalian proteins, has been reported to trigger delayed severe allergic reactions to red meat and to induce acute allergic reactions upon injection of therapeutic antibodies carrying α-gal (20, 21). Galacto-oligosaccharides (GOS) are heat-stable, non-digestable carbohydrates present in milk formula and as supplement in different beverages. Food allergy to GOS are reported in Singapore, Vietnam and Japan (19). The structure and characterization of carbohydrates that are the reported targets of IgE binding have not been demonstrated, although they have been assumed to be related to the synthesis of GOS complexes. Recent work failed to identify the structures or link them to activation of basophils (22). In contrast to α-gal, the GOS carbohydrates have not been linked to a protein backbone (19). So far, only allergens representing protein epitopes have been named by the WHO/IUIS Nomenclature Sub-Committee. The committee is currently considering whether there is enough definition of carbohydrate epitopes to provide unique names and whether names would be scientifically useful.

### Updating Existing Allergen Entries Named From Partial Allergen Sequences

Some allergens are characterized based on peptide sequences from native molecules. In some cases, full-lengh sequences were not available, but are based on peptide matches to genomic sequences. That was the case for fish allergens, enolase Sal s 2 and aldolase Sal s 3 from salmon (*Salmon salar*) based on public genomic sequences (23). For the salmon allergens Sal s 2 and Sal s 3, complete sequences were attributed without testing of the allergenic properties of the corresponding proteins. In the future, we recommend that an alignment of the complete sequences of natural or recombinant protein be used to determine IgE binding be compared to the genome sequence, or at the least, a test of the recombinant protein be comparison to the native counterpart. Importantly, different isoforms and variants can exist in the same source and although highly similar, those proteins can differ in their allergenic potency (24). Verified information will be added to the existing database entry in order to further improve its quality.

### Numbering of Allergens

The allergen nomenclature system was originally based on the order of allergen discovery to provide the allergen number, but the commitee recognized the conservation of sequences and structures are important. But sometimes, very similar allergens from related species receive different numbers (e.g., Fel d 2 and Can f 3) (25). It would be disruptive to change allergen names after a number of publications have used established allergen names and only allergen numbers have been revised and changed in a few cases (26).

## Overview of Outdoor Environmental Aeroallergens

Most outdoor aeroallergens are derived from grass, weed and tree pollen or fungal spore sources. Although Blackley discovered that pollen caused hay fever in the nineteenth century (27), it was not until allergenic fractions were purified by chromatography from ryegrass pollen in the 1960s (28) and cloned in 1990s (29) that the first pollen allergens were characterized. Because the sources are typically not recognized due to small sizes of pollen (10–50 microns) and mold spores (2–50 microns), there is often uncertainty regarding the causal link between exposure to a given species and allergic reactions. This is specifally difficult given the cross-reactivities that exists among allergens from different species.

The IUIS Allergen Nomenclature Subcommittee recognize 43, 55, and 45 allergens for pollen of grasses, trees and weeds, respectively. Another four pollen allergens of herbaceous plants included; Lig v 1 of *Ligustrum vulgare* (Common privet), Syr v 1 and Syr v 3 of *Syringa vulgaris* (Lilac) and Hum j 1 of *Humulus japonicus* (Japanese hop).

Grass pollen allergens belong to 12 different protein families. The most abundant proteins of mature pollen that elicits a high frequency of IgE reactivity are the major group 1 beta-expansin allergens. The purpose of beta-expansins is to degrade cell walls and allow pollen tube extension which is essential for fertilization. A number of IgE and T cell epitope regions are found in close proximity with the enzymatic domain and specifically the HFD motif of the N terminal domain (30–32). While allergens of temperate grass pollens were characterized in 1990s, allergens of subtropical grass pollens continue to emerge. Grass pollen group 1 allergens among the Pooideae temperate grasses share between 84 and 91% amino acid identity, while subtropical grasses of Panicoideae, Chloridoideae, and Oryzoideae share between 49 and 86% identity (Table S1). Separate gene loci encode multiple Poaceae group 1 isoforms. The pollen expansin proteins show significant sequence diversity, but they apparently share similar functions exemplified by Cyn d 1 and Sor h 1 isoform diversity (Table S1). While research shows that there is IgE and T cell cross-reactivity between group 1 and group 5 grass pollen allergens across broad taxa, their heterogeneity contributes to the diversity of immune recognition (33, 34). The most recently listed (grass pollen allergen is Uro m 1 of the subtropical Panicoideae grass *Urochloa mutica* (Para grass). Group 2 and 3 grass pollen allergens share approximately 30% identity with the carboxyl domain of the beta-expansin protein family. Consequently, grass pollen group 2/3 pollen allergens are smaller in size (10–12 kDa), but their function is unknown. Sor h 2 and Ory s 2 are described in subtropical species, but their clinical importance is less well-demonstrated. Several isoforms and multiple variants exist for Group 5 allergens (e.g., for Phl p 5) and these share between 53 and 78% identity between six species for which these allergens have been characterized (Table S2). Group 5 allergens are absent from subtropical grass pollen.

Weed pollen allergens arise from 16 species including ragweed (*Ambrosia artemisiifolia*), mugwort (*Artemisia vulgaris*), pellitory (*Parietaria judaica*), amaranth, thistle (*Salsola kali*), and sunflower (*Helianthus annuus*) and belong to 10 allergen families. Clinically important allergens of the Asteraceae family include pectate lyases (Amb a 1 and Art v 6), defensin-like proteins (Amb a 4 and Art v 1), non-specific lipid transfer proteins (Art v 3 and Par j 2), whereas allergens of the Amaranthaceae family include Ole e 1-like proteins and Che a 1, and pectin methylesterase (Sal k 1).

Trees from multiple orders; Fagales; for example, Birch (*Betula verrucosa*), Lamiales; Ash (*Fraxinus excelsior*) and Olive (*Olea europaea*), Proteales; Plane (*Plantanus acerifolia*) and Cupressales; Japanese Cedar (*Cryptomeria japonica*), encompassing 21 species contribute allergens belonging to 18 protein families. Clinically important allergens include Pathogenesis Related protein-10 (Bet v 1, Ole e 1-like protein); Fra e 1, polygalacturonase; Cry j 2 and Pla a 2, and pectate lyase; Cry j 1. Profilins are ubiquitous and highly conserved in sequence and structure (e.g., Phl p 12, Amb a 8 and Bet v 2) and polcalcins (Phl p 7, Amb a 9, and Ole e 8) are pan-allergens common to grass, weed and tree pollen, though these allergens are generally less potent than PR-10 proteins.

A variety of fungi produce the 112 listed WHO/IUIS allergens from as many as 36 protein families. While fungal hyphae or spores may be used as food or drug sources, many are common sources of indoor and outdoor allergy and a small number colonize human tissues including skin and lungs. Spores of 15 Ascomycota species belonging to diverse fungal families; Aspergillaceae, Cladosporiaceae, Didymellaceae, Nectriaceae, and Pleosporaceae produce 72 airborne outdoor fungal spore allergens. There are 23 allergens listed for *Aspergillus fumigatus* including the ribotoxin (Asp f 1), peroxisomal protein (Asp f 3), metalloprotease (Asp f 5), cyclophilin (Asp f 11), vacuolar serine protease (Asp f 18), and enolase (Asp f 22), which are all major allergens. *Alternaria alternata* spores, one of commonly recognized fungal spores contains 12 listed allergens including its major allergens (Alt a 1), and, a glutathione S transferase (Alt a 13).

## Overview of Occupational Allergens From Food Sources

Occupational sensitization has been observed for centuries in historical and medical writings. The first description of baker's asthma was probably by Ramazini around 1700; the first report of allergic responses to fish was in 1937 by De Besche relating to a fisherman who developed allergic symptoms when handling codfish (35). Sensitization to allergens at the workplace occurs usually via the skin or the respiratory tract. Resulting cutaneous and respiratory diseases are a global health problem, with an estimate that 25% of all asthma cases related to work (36).

The most common workplace allergens are relatively high-molecular-weight proteins derived from cereal flour, natural rubber latex, wood dust, livestock and laboratory animals, seafood, industrial enzymes and mold (37). Animal and vegetable high-molecular-weight proteins present in the aerosol and often in aerosolized foods during food processing are the main inhalant allergen sources. This type of IgE-mediated respiratory food allergy usually does not generate any symptoms upon ingestion of the source material and is suggested to reflect a new type of allergy, termed Class 3 food allergy (37).

Over 400 occupational allergenic sources are identified and documented as sensitizers, derived from plants, animals and microbes. However, few have been characterized on the molecular level and only a limited number of purified native or recombinant allergens are available for testing (37). The current WHO/IUIS database categorizes allergens by typical route of exposure. However, in the case of occupational allergens the specific route is often not clear, and the route of sensitization may differ from the common route of elicitation and may be listed as “unknown.”

A good example of the complex nature of identifying “occupational allergens” among plants is wheat (*Triticum aestivum*), where 28 allergens are registered with the WHO/IUIS, however only 13 are confirmed food allergens. The route of sensitization is categorized as “unknown” for 15, with 14 being relevant in baker's asthma and several allergens are relevant for grass-pollen cross-reactivity, including profilin (Tri a 12) and a subunit of the tetrameric heterologous a-amylase inhibitor chloroform/methanol-soluble CM17 protein (Tri a 40).

Among animal derived allergens, tropomyosin is an abundant and clear food allergen from crustacean shellfish and molluscs that is also implicated in IgE binding and possibly in triggering airway allergy from mites and some insects. *In vitro* IgE binding studies and skin tests without clear clinical histories of the patient can lead to uncertain diagnosis due to high amino acid homology across diverse taxonomic groups. Clear differentiation is often only possible if IgE inhibition studies are performed and verification of biological activity. Dose and exposure are important. The relevance of IgE binding to environmental sources (house dust mite, cockroach and other organisms) often leads to speculation about route of exposure in sensitization. Of the 35 registered tropomyosins, 22 are known food allergens, while 13 are not known to cause food allergies. Of these 10 are derived from insects or mites and the route of sensitization is most likely via inhalation. Among the food allergens 19 tropomyosin are derived from different seafood species and are relevant occupational allergens, however not identified as such (38). Some animal allergens are not commonly recognized by patients or clinicians due to limited exposure for most people to the source. Hemoglobins from the insect “harlequin fly larvae,” *Chironomus thummii* bind IgE from sera of those who raise the insects for fish food (39). A few recent cases of occupational allergy have been noted for workers who raise mealworms (*Tenebrio molitor*) as a source of protein for processed human food (9, 40). Some individuals have been newly sensitized at work, while for others it appears, they were sensitized by consumption of shrimp or other crustaceans. Highly conserved tropomyosin and a few enzymes such as arginine kinase may be responsible for cross reactivity.

Many other foods have been reported in single case reports as the cause of occupational allergies and asthma, and sensitization by inhaled allergen exposure is therefore quite likely (41). Importantly, though these proteins may be tolerated during exposure by one route or by most people, they may not be tolerated if exposure occurs differently and as such, the WHO/IUIS nomenclature committee has not asked for *in vivo* proof that new allergens illicit allergic responses to date, only *in vitro* evidence and a good clinical history of symptoms.

## Summary and Conclusions

Though molecular and scientific advances have led to improved characterization of allergens and with-it increased clarity in naming and categorizing them, the heterogeneous nature of human data remains a challenge. This Sub-Committee has in the past asked for minimal data characterizing protein sequences, clinical symptoms of serum donors or test subjects, IgE binding and allergic functionality. We see great diversity in data submitted with candidate proteins. Our recent publications are intended to help researchers focus on relevant questions to improve the evidence that individual proteins are allergens, and not simply cross-reactive in IgE binding with only mild clinical consequences. Sequences of the allergens and demonstration of IgE binding from relevant symptomatic individuals are essential for defining allergens. Some researchers would like to simply submit to the Sub-Committee information about recombinant proteins based only on genomic sequences of potential allergen sources. While that activity may help predict possible allergens, our goal is to call for direct proof of allergenicity using subjects with clear clinical reactivity to source materials, and with clear IgE binding to proteins presented at representative concentrations.

The purpose of the allergen nomenclature system is to help researchers, clinicians, pharmaceutical companies, regulators, and the public clearly understand the identity of clinically important allergens for diagnosis and to help ensure compliance for improved safety.

However, our system is not perfect. Recent research shows that some specific glycans are important allergens including α-gal that is bound to tick and mammalian proteins and to glycol-lipids. We do not define these structures as allergens but hope to help publicize risks and educate consumers and allergists about risks.

## Author Contributions

Each author contributed a significant portion of writing for the manuscript and reviewed the final document. This collection of authors is a representative segment of the WHO/IUIS Allergen Nomenclature Sub-Committee. AK is not a Sub-Committee member, but has contributed to identification and characterization of allergens in this manuscript.

### Conflict of Interest

AP is employed by the company Indoor Biotechnologies, Inc. The remaining authors declare that the research was conducted in the absence of any commercial or financial relationships that could be construed as a potential conflict of interest.
