# Supplementary material for: Keeping Allergen Names Clear and Defined
Source: Front Immunol. 2019 Nov 19;10:2600. doi: 10.3389/fimmu.2019.02600 (PMC6878850; doi:10.3389/fimmu.2019.02600)
Supplement: Supplementary file 1 [file Data_Sheet_1.docx]

Table S1. Percentage identity of grass pollen beta-expansin group 1 allergens across group 1 allergens using Clustal 2.1.

| Poaceae group 1 pollen allergens* | | 1 | 2 | 3 | 4 | 5 | 6 | 7 | 8 | 9 | 10 | 11 | 12 |
| --- | --- | --- | --- | --- | --- | --- | --- | --- | --- | --- | --- | --- | --- |
| 1 | Sor h 1.0201 | **100** | 82 | 81 | 49 | 63 | 62 | 54 | 55 | 54 | 52 | 54 | 53 |
| 2 | Pas n 1.0101 | 82 | **100** | 84 | 54 | 65 | 64 | 60 | 60 | 60 | 59 | 59 | 58 |
| 3 | Zea m 1.0101 | 81 | 84 | **100** | 58 | 66 | 63 | 56 | 57 | 59 | 56 | 56 | 55 |
| 4 | Ory_s 1.0101 | 49 | 54 | 58 | **100** | 60 | 61 | 59 | 61 | 65 | 63 | 63 | 65 |
| 5 | Cyn_d 1.0101 | 63 | 65 | 66 | 60 | **100** | 86 | 66 | 68 | 69 | 69 | 66 | 65 |
| 6 | Cyn_d 1.0201 | 62 | 64 | 63 | 61 | 86 | **100** | 68 | 69 | 70 | 72 | 69 | 68 |
| 7 | Sor_h 1.0101 | 54 | 60 | 56 | 59 | 66 | 68 | **100** | 69 | 71 | 71 | 68 | 69 |
| 8 | Hol_l 1.0101 | 55 | 60 | 57 | 61 | 68 | 69 | 69 | **100** | 88 | 91 | 89 | 90 |
| 9 | Phl p 1.0101 | 54 | 60 | 59 | 65 | 69 | 70 | 71 | 88 | **100** | 91 | 84 | 86 |
| 10 | Poa p 1.0101 | 52 | 59 | 56 | 63 | 69 | 72 | 71 | 91 | 91 | **100** | 87 | 89 |
| 11 | Pha a 1.0101 | 54 | 59 | 56 | 63 | 66 | 69 | 68 | 89 | 84 | 87 | **100** | 90 |
| 12 | Lol p 1.0101 | 53 | 58 | 55 | 63 | 65 | 68 | 69 | 90 | 86 | 89 | 90 | **100** |

*One representative isoform for allergens for which complete protein sequences are available from Subtropical (Panicoideae (row 1-3 and 7); Chloridoideae (row 5-6); and Oryzoideae (row 4) and temperate (Pooideae(row 8-12) grasses. Percentage identity matrix calculated by EMBL Clustal 2.1.

Table S2. Percent identity of grass pollen group 5 and 6 allergens of different species using Clustal 2.1.

| Poaceae group 5 pollen allergens* | | 1 | 2 | 3 | 4 | 5 | 6 | 7 | 8 |
| --- | --- | --- | --- | --- | --- | --- | --- | --- | --- |
| 1 | Phl p 6.0101 | **100** | 46 | 47 | 63 | 51 | 57 | 56 | 59 |
| 2 | Pha a 5.0101 | 46 | **100** | 54 | 63 | 57 | 59 | 53 | 60 |
| 3 | Sec c 5.0101 | 47 | 54 | **100** | 64 | 57 | 56 | 64 | 66 |
| 4 | Hol l 5.0101 | 63 | 63 | 64 | **100** | 72 | 77 | 69 | 74 |
| 5 | Poa p 5.0101 | 51 | 57 | 57 | 72 | **100** | 78 | 60 | 66 |
| 6 | Phl p 5.0201 | 57 | 59 | 56 | 77 | 78 | **100** | 61 | 65 |
| 7 | Lol p 5.0101 | 56 | 53 | 64 | 69 | 60 | 61 | **100** | 77 |
| 8 | Phl p 5.0101 | 59 | 60 | 66 | 74 | 66 | 65 | 77 | **100** |

*One representative isoform for allergens for which protein sequences are available are included. Percentage identity matrix calculated by EMBL Clustal 2.1.
